# Acute emergency tibialization of the fibula: reconstruction of a massive tibial defect in a type IIIC open fracture

**DOI:** 10.1007/s11751-013-0167-6

**Published:** 2013-07-27

**Authors:** Fatih Parmaksızoğlu, Eren Cansü, Mehmet Bekir Ünal, A. Yener Ince

**Affiliations:** 1 Yeni Yüzyil University, Istanbul, Turkey; 2 Marmara University, Tütüncü Mehmet Efendi Cd. No:28/12 Göztepe, Istanbul, Turkey; 3 Medipol University, Istanbul, Turkey; 4 Universal Hospital Camlica, Istanbul, Turkey

**Keywords:** Acute tibialization, Emergency tibialization, Tibial defect, Type IIIC open tibial fracture, Lower-extremity reconstruction

## Abstract

Gustilo type IIIC open fractures of the tibia are high-energy injuries necessitating long treatment periods and usually multiple surgical procedures and eventually resulting in high morbidity rates and even amputations. We present here a case involving a type IIIC open tibial fracture with massive loss of the entire tibial diaphysis, which we treated by performing acute tibialization of the fibula after revascularization of the posterior tibial artery in a single-stage emergency operation.

## Introduction

Severe segmental tibial bone loss occurring after an open fracture is a considerable challenge to the treating orthopedic surgeon. Amputation is a difficult decision as it is an irreversible procedure in this limb-threatening injury. On the other hand, reconstruction of the injured limb requires both replacement of the bony defect and soft tissue repair. A number of techniques have been described for salvage of the injured limb, including serial massive debridement and early flap closure, autologous bone grafting, vascularized or non-vascularized transfer of the ipsilateral or contralateral fibula, segmental transposition, tibiofibular synostosis, and acute shortening and relengthening.

We report a case of a Gustilo type IIIC open fracture with massive loss of the tibial diaphysis. After the damaged, tibialis posterior artery was repaired at the level of the ankle during emergency surgery, and immediate intramedullary insertion of the ipsilateral fibula on its peroneal arterial pedicle between the two preserved edges of the tibia was performed in a single procedure. To our knowledge, this is the first case in which emergency single-stage acute tibialization of the fibula is described.

## Case report

**Fig. 1:**
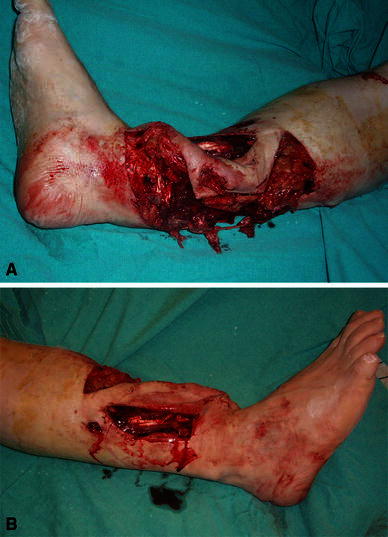
**a**, **b** Massive injury to the tibia and the surrounding soft tissue

**Fig. 2:**
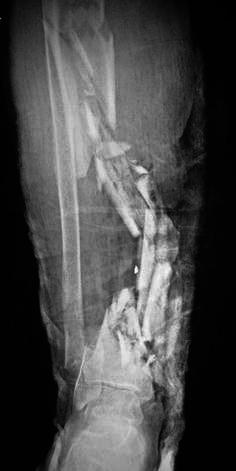
Although nearly the entire tibial diaphysis had been destroyed, the fibula remained intact along its total length, except at its distal pole

**Fig. 3:**
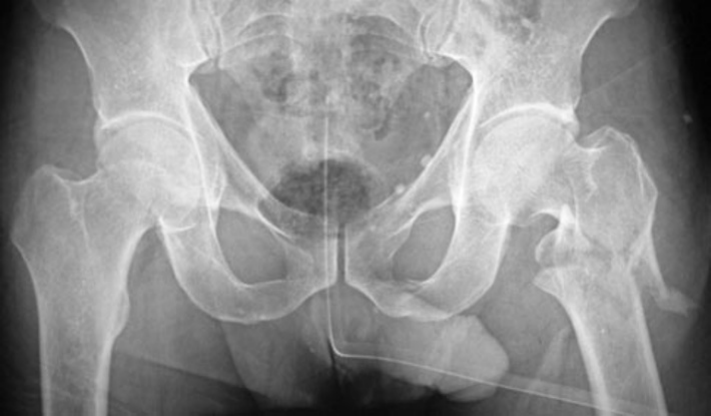
Preoperative radiograph of the fractured left femur

**Fig. 4:**
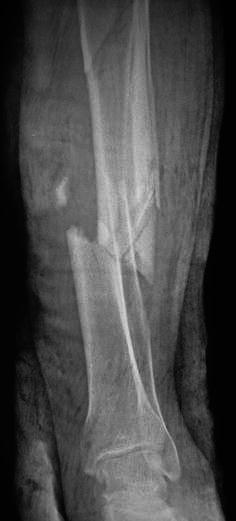
Preoperative radiograph of the fractured left tibia

In a motor vehicle accident, a 42-year-old man sustained several injuries: a Gustilo type IIIC open fracture of the right tibia; an intertrochanteric fracture of the right femur; an uncomplicated left tibial and fibular fracture; and a calcaneal skin defect on his left side (Figs. 1, 2, 3, 4). He had an ischemic limb due to an arterial injury at the ankle on the right side.

The patient was taken immediately to the operating room and anaesthetised. All devitalized bony and soft tissues were excised on the side of the open tibial fracture. The tibial bone defect was nearly 25 cm after debridement, and the remaining two ends of the tibia were stabilized with a unilateral fixator. Two Schanz pins were placed in the proximal part of the tibia, and one each was placed in the distal tibial end and in the talus. The tibial nerve was compressed but intact at the ankle. At the same level, the posterior tibial artery was also compressed and thrombosis detected in a 1-cm segment. The thrombotic segment was excised and end-to-end primary arterial re-anastomosis was performed to revascularize the distal lower extremity. It was decided to manage the bone defect by transferring the ipsilateral fibula which was entirely intact but for a simple fracture at the level of the ankle joint.

**Fig. 5:**
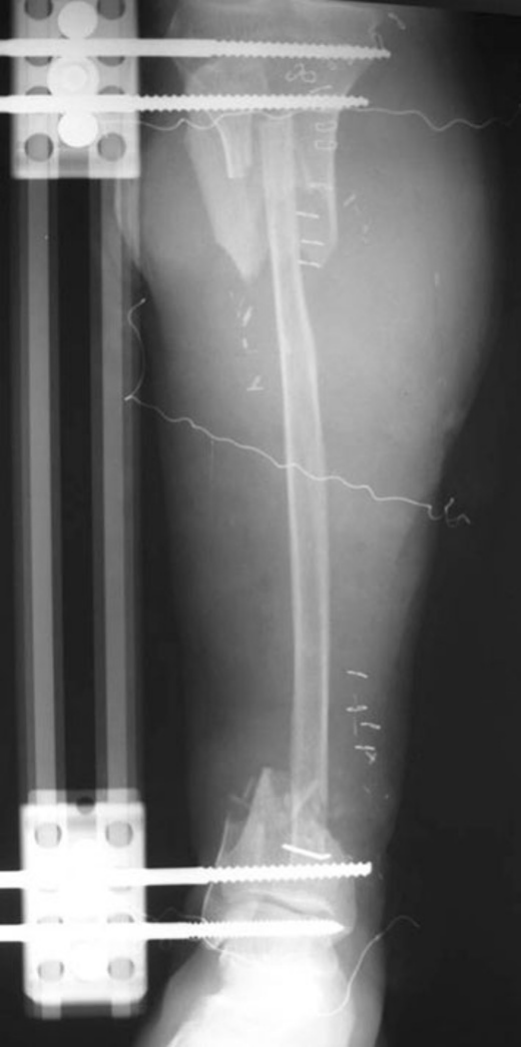
An early postoperative radiograph shows that the fibula is seated well between the two poles of the tibia

The fibula was exposed and then osteotomized proximally just distal to the neck. The length of the fibula transferred was nearly 30 cm. To facilitate transfer of the fibula, a careful dissection was performed. The attached soft tissue and peroneal arterial supply were protected. After preparation of the remaining tibial ends, the vascularized fibula was transferred medially and placed between the two ends of tibia. During that transfer, placement of the unilateral fixator was adjusted to obtain gross overall alignment (Fig. 5). The soft tissue defect was covered using remaining undamaged skin with additional skin grafting for uncovered areas. There was no postoperative infection.

The other injuries were treated as follows: proximal femoral nailing for the intertrochanteric fracture; intramedullary nailing for the left tibial fracture; and placement of a propeller flap for the calcaneal skin defect. These were performed at 2 weeks once it was declared the right side had no evidence of infection. At the end of the third week, a valgus deformity of the transferred segment of fibula was corrected by realigning the distal Schanz pins under fluoroscopy.

**Fig. 6:**
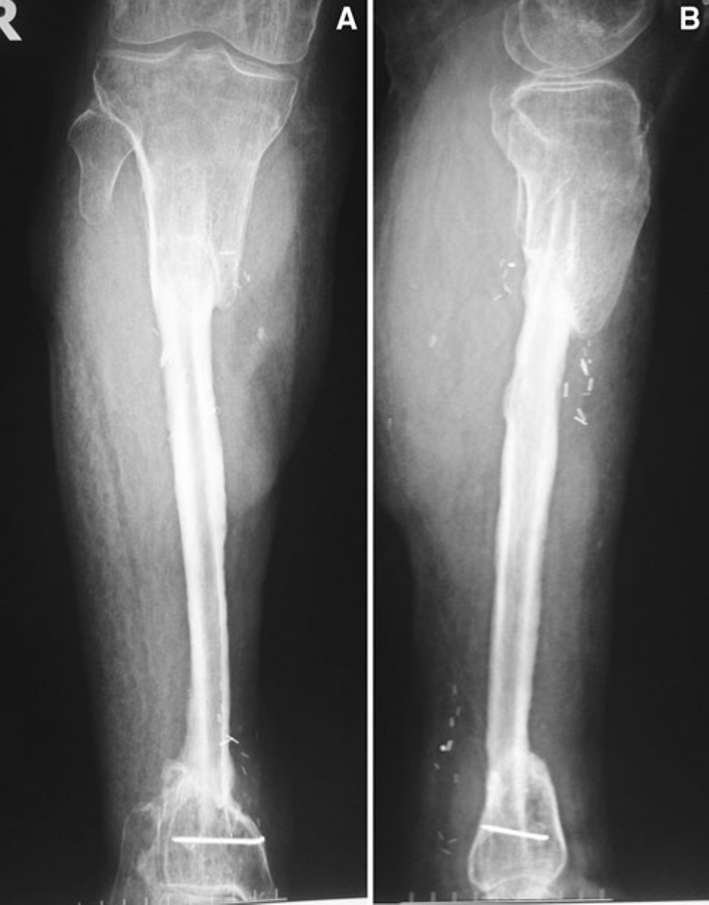
**a**, **b** Radiographs obtained 2 years after surgery show that the transferred fibula has been fully consolidated to allow full weight bearing. Thickening is well demonstrated

At review at 5 months, during which the intervening follow-up period was without complication, the state of bone union was evaluated and the patient was encouraged to engage in partial weight bearing with crutches. At 8 months, the patient was able to walk with a single crutch and without crutches at 14 months. At the 2-year follow-up examination, hypertrophy of the fibula was apparent (Fig. 6a, b). Complete union was achieved, and the patient did not experience refracture, infection, or permanent pain. Sensation in the sole of his affected foot had recovered and he did not require additional pain medication. There was a limb length discrepancy of 2.5 cm. At the end of the treatment that did not produce an obvious limp. Although an insole was prescribed, it was not used by the patient for most of the day.

## Discussion

Open fractures of the tibia accompanied by massive segmental bone loss and critical vascular injury are major treatment challenges. Primary amputation is still a common choice in several centers but when the circulation of foot is reestablished and the sensation of the sole is preserved, limb-salvage procedures are an option. The total cost of the procedure may appear, after cursory assessment, to be three to four times that of amputation, but in fact, it is much more economical when the long-term outcome is considered [1–3].

Conventional bone grafting is useful for repairing defects smaller than 5 cm in cases where there is sufficient vascularization and no infection. Microsurgical techniques may make it possible to treat larger defects through use of free vascularized bone grafts; vascularized bone from the fibula or iliac crest are potential solutions for larger defects, but the iliac crest can be a donor source only for defects of a maximum length of 10–15 cm [4]. The anatomic features of iliac bone also present a congruency problem for the replacement of tubular bone. To avoid these difficulties, vascularized fibular graft has been used extensively for reconstruction. The contralateral fibula is considered an ideal source of free vascularized bone graft, but the integrity of the donor fibula is essential in this procedure [4].

In recent years, external fixators are used widely for this type of bone reconstruction. The recent modifications to external fixators and the concept of segmental fragment transfer by distraction osteogenesis have provided the surgeon with various alternatives for dealing with these compound injuries. Internal segmental bone transfer and acute shortening and relengthening techniques are the main strategies for the treatment of tibial bone defects with an external fixator. However, these methods are limited by the sufficiency of bone reserve [1, 5–10] and disadvantaged by a long treatment duration [1].

Another method for repairing larger defects is the transfer of ipsilateral fibula to consolidate the fibula and tibia, called tibialization of the fibula. After a proximal and distal fibular osteotomy, the whole fibular graft can be transferred gradually using the Ilizarov method to bridge a tibial defect. Traction with olive wires and subsequent gradual transfer is the main principle of the method [11–19]. After the transfer, reconstruction is achieved with eccentric tibiofibular synostosis, secured with grafting techniques [11–14, 20, 21].

In this patient, the defect was too large to be repaired by a simple conventional bone graft or by a vascularized flap from the iliac crest. In addition, a contralateral comminuted fracture interfered with our ability to use the other fibula as a vascularized graft, and it was impossible to perform an internal segmental bone transfer or acute shortening and relengthening as, after debridement of devitalized tissues, the remaining tibial bone stock was poor.

Despite an extremely fragmented tibia with large bone defect, this patient had a well-protected ipsilateral fibula which was fractured only a few centimeters from the distal end. During surgery, we observed that the proximal connection of the peroneal artery was intact and decided that transfer of this large vascularized fibula segment would be an ideal alternative for replacing lost tibial bone. After preparation of the two ends of the tibia, we transferred the vascularized fibula medially and placed it in between the two poles of the tibia. Our preferred fixation method with a monolateral external fixator does not allow early weight bearing before callus formation. Conversion to a circular frame after the soft tissues have healed would have been an alternative which is more stable. Although referred to in the literature, we did not experience complications of pin loosening and secondary deformities in this patient or in our previous patients to whom we performed limb lengthening procedures with monolateral external fixators [1].

Stress fracture of the fibular graft is not an uncommon complication of this technique and was reported between 7 and 35 % in the literature [22–25]. This graft did not fracture in the period of follow-up.

An analysis of the literature reveals that medial transfer of the ipsilateral fibula has been performed mostly as a secondary procedure involving gradual transfer [6, 8, 9, 17] or, in a few cases, as a secondary immediate procedure [7]. These cases generally involve high-energy trauma and always entail large soft tissue and bony–tissue defects. During the healing period, tissue repair and fibrosis impair the normal regional anatomy and interfere with proper vascular microsurgical dissection. Delayed surgery would likely be more challenging because of the difficulty of neurovascular dissection in an area with adhesive and fibrotic tissue. Therefore, we believe that immediate fibula transfer is preferable, as a first therapeutic step, to delayed surgery in selected cases.

## Conclusion

Acute transfer of the ipsilateral fibula with its vascular pedicle intact should be considered an option for the management of large tibial defects when techniques of bone transport or acute shortening/lengthening are deemed too risky or entail an excessively prolonged treatment period.
